# New Insight in Pediatric Orthopedic Oncology: The Use of a Xeno-Hybrid Bone Substitute in Loss of Bone Tissue After Oncological Resections, a Case Series

**DOI:** 10.3390/jcm15062329

**Published:** 2026-03-18

**Authors:** Raimondo Piana, Raffaella De Pace, Michele Boffano, Carlo F. Grottoli, Nicola Ratto, Pietro Pellegrino, Maria Chiara Rossi, Giuseppe Perale

**Affiliations:** 1 Department of Orthopaedic Oncologic Surgery, Orthopedic Trauma Center (CTO) and Regina Margherita Hospital (OIRM), University Hospital (AOU) Citta’ della Salute e della Scienza of Turin, Via Zuretti 29, 10126 Torino, Italy; 2 Department of Chemical, Pharmaceutical and Agricultural Sciences, University of Ferrara, 44121 Ferrara, Italy; 3 Industrie Biomediche Insubri SA, via Cantonale 67, 6805 Mezzovico-Vira, Switzerland; 4 Faculty of Biomedical Sciences, University of Southern Switzerland (USI), Via G. Buffi 13, 6900 Lugano, Switzerland; 5 Ludwig Boltzmann Institute for Experimental and Clinical Traumatology, Donaueschingenstrasse 13, 1200 Vienna, Austria

**Keywords:** pediatric bone tumors, xenohybrid bone substitute, surgical reconstruction, bone regeneration, osteointegration

## Abstract

**Background**: The management of bone defects in pediatric oncology represents a major challenge in orthopedics, as it requires preserving both joint function and skeletal growth. Traditional reconstructive approaches, such as autografts and allografts, are limited by availability, complications, and incomplete biological integration. In this context, xeno-hybrid bone substitutes have emerged as a promising alternative. The aim of this study was to evaluate the safety and effectiveness of SmartBone^®^ ORTHO in the reconstruction of post-oncological bone defects in children. **Methods:** Twelve pediatric patients treated at the Centro Traumatologico Ortopedico (CTO) and OIRM Hospital, AOU Città della Salute e della Scienza of Turin (Italy), between 2016 and 2019 were retrospectively analyzed. Lesions included simple and aneurysmal bone cysts, non-ossifying fibroma, chondroblastoma, and other benign conditions. All patients underwent curettage followed by defect filling with SmartBone^®^ ORTHO. **Results:** At clinical and radiological follow-up, nine patients (75%) showed stable graft integration and complete functional recovery. Three patients (25%) developed local recurrence, which was managed with revision surgery and re-implantation of SmartBone^®^, with all achieving stable outcomes. Radiographs demonstrated progressive increases in bone density and trabecular thickness, reaching values comparable to those of native bone within 6–12 months. **Conclusions**: SmartBone^®^ ORTHO proved to be a safe and effective biomaterial for pediatric post-oncological bone reconstruction, promoting rapid osteointegration and physiological bone remodeling without infection or intolerance.

## 1. Introduction

Bone tumors are a rare and heterogeneous group of neoplasms that develop within bone tissue. The fifth edition of the 2020 WHO Classification of Soft Tissue and Bone Tumors provides an updated classification scheme based on the type of tissue of origin and the biological behavior of the lesions [1]. Osteogenic tumors originate from bone-forming tissue. Among the benign types are osteoma, osteoid osteoma, and osteoblastoma, which are characterized by slow growth and lack of metastatic potential. The main malignant osteogenic tumor is osteosarcoma, known for its aggressive behavior and high risk of metastasis [2,3]. Chondrogenic tumors develop from cartilaginous tissue. Benign variants include chondroma, enchondroma, osteochondroma, chondroblastoma, and chondromyxoid fibroma. The most relevant malignant form is chondrosarcoma, whose clinical behavior varies depending on the degree of differentiation of its constituent cells [2,4]. Fibrogenic tumors arise from fibroblastic tissue. Among them, non-ossifying fibroma and chondromyxoid fibroma are benign, although they may occasionally exhibit locally aggressive growth. Fibrosarcoma of bone, on the other hand, is a rare malignant neoplasm with high infiltrative and metastatic potential [2,5]. Giant cell tumors of bone are characterized by abundant osteoclast-like giant cells. They are classified as benign but locally aggressive, with a tendency to recur [2,6]. Aneurysmal bone cysts (ABC) are considered benign pseudotumoral bone lesions. They are not true neoplasms but rather expansive osteolytic lesions made up of blood-filled cystic spaces separated by septa of connective tissue containing multinucleated giant cells, osteoclasts, and osteoid reactions. Despite their benign classification, ABCs can be locally aggressive, leading to bone deformities, pain, and a risk of pathological fractures. They primarily affect children and adolescents [2,7]. Simple bone cysts are also regarded as benign, non-neoplastic pseudotumoral lesions according to the WHO 2020 classification. They are non-aggressive, typically asymptomatic, and often discovered incidentally during childhood or adolescence. Although benign, these cysts may structurally weaken bone and increase the risk of pathological fractures, particularly during growth phases [2].

In bone tumors, bone loss must be addressed as a surgical reconstruction challenge. In benign tumors, bone loss often results from an intraosseous mass following curettage or excision. Curettage can be combined with adjuvant treatments in aggressive benign lesions, such as giant cell tumors of bone or aneurysmal bone cysts, to reduce the risk of local recurrence [8]. More recently, these adjuvant agents have been replaced by thorough burring of the curetted margins, often combined with disease-control therapy, such as denosumab, in more sensitive lesions. In malignant cases, bone loss reconstruction can be more complex. Bone graft substitutes can be considered valuable tools to complement surgical intervention in these challenging cases. Moreover, they can also be used to treat complications arising from failed bone reconstructions in more demanding tumor cases [8,9].

Bone substitutes are increasingly being used in surgery, with over two million bone grafting procedures performed worldwide each year. They are particularly utilized in oncologic surgery, traumatology, revision prosthetic surgery, and spine surgery. The ideal bone substitute should be biocompatible and should not trigger any adverse inflammatory response. It should be easily shaped to fit the bone defect within a short setting time [10]. Additionally, it should be osteoconductive, osteoinductive and resorbable. The grafted bone can be easily colonized by the host’s bone cells and progressively replaced by newly formed bone. It serves as an excellent scaffold for rapid stabilization and bone reconstruction, being second only to autologous bone for this purpose [11].

Although autologous bone grafting is still considered the “gold standard” for repairing bone defects, its availability is limited, and its harvesting can be painful and may lead to local complications for the patient. In response to these challenges, significant progress has been made over the past century in developing viable alternatives. Bio-banked allograft bone represents a suitable alternative to autologous bone, as it is also derived from human donors. While donor bone is osteoconductive, it is only weakly osteoinductive [12]. Furthermore, allografts often require sterilization, which can negatively impact their mechanical and biological properties. Concerns have also been raised regarding potential infectious risks. Bovine xenografts are valid scaffolds for bone grafting, as they closely resemble human cancellous bone. However, the sterilization process may alter their biomechanical properties. Recently, low-temperature processing of bone and the incorporation of composite technology into xenografts have shown promising results in improving their biological and mechanical performance in clinical practice [12].

SmartBone^®^ ORTHO (Industrie Biomediche Insubri IBI SA, Mezzovico-Vira, Switzerland) is a composite xeno-hybrid graft obtained from a low-temperature processed bovine-derived mineral matrix extracted from the internal part of the femoral heads of adult bulls. It is enriched with synthetic aliphatic polyester poly(L-lactide-co-ε-caprolactone) (PLCL) and Arg-Gly-Asp (RGD)-containing collagen fragments (derived from animal gelatin). This combination enhances elasticity, blood affinity, and cell attachment while providing an open, porous, and interconnected microenvironment (with an average pore size of 250 microns) that facilitates cell and vessel colonization, ultimately allowing for complete remodeling over time [13].

Pediatric bone oncology represents one of the most complex challenges in orthopedic surgery. Bone tumors often require invasive surgical treatments, which frequently involve extensive bone resections. This type of intervention can result in significant bone tissue loss, with the risk of compromising the function of the affected limb and the skeletal growth of young patients. In this context, reconstructive surgery plays a crucial role, aiming to preserve the limb and restore physical function while minimizing the risks of post-operative complications [14]. Treating bone tumors in young children is challenging because of the need to preserve growth potential, maintain joint function, and conserve bone for future revisions [15], yet even if increasing such pathologies still remain rare.

Reconstructive options following tumor resection include the use of bone grafts, designed to replace the removed bone segment, ensuring structural and functional support. The choice of technique depends on various factors, including the patient’s age, the tumor’s location, and the general health condition of the patient [16].

This study aims to explore new frontiers in pediatric surgery within orthopedic oncology, with a particular focus on the use of xeno-hybrid bone substitutes for managing postoperative bone tissue loss. Specifically, the goal is to evaluate the effectiveness and clinical outcomes of the xeno-hybrid bone substitute SmartBone^®^ ORTHO in treating bone loss in pediatric oncology patients. The study examines the reconstruction of bone defects caused by both benign and malignant bone tumors, documenting the application of this innovative biomaterial in 12 pediatric cases.

## 2. Results

### 2.1. Clinical and Radiological Outcomes

A total of 12 pediatric patients underwent surgical treatment for benign bone lesions with reconstruction using the xenohybrid bone graft SmartBone^®^ ORTHO. In 9 cases (75%) the clinical and radiological outcomes were fully satisfactory, with no evidence of recurrence or postoperative complications during follow-up. Three patients (25%) developed local pathology recurrence: one child with an aneurysmal bone cyst of the proximal tibia experienced relapse and underwent repeat curettage with reimplantation of SmartBone^®^, achieving stable integration; a patient with a simple bone cyst of the proximal humerus had recurrence treated with revision curettage and allograft; and another case concerned a chondroblastoma of the proximal humerus that relapsed and was successfully managed with a second curettage and SmartBone^®^ reimplantation.

Radiological evaluation demonstrated consistent osteointegration and progressive remodeling of the xenograft in the majority of cases. Early signs of bone regeneration, such as increased density and trabecular reorganization, were already evident one to two months after surgery. These changes continued to progress over three to six months, with complete incorporation and remodeling comparable to the adjacent native bone typically achieved within six to twelve months. This remodeling pattern confirmed the osteoconductive properties of the scaffold and its successful integration within the host bone.

#### 2.1.1. Case Series Results

##### Case ONCO-001

**Figure 1:**
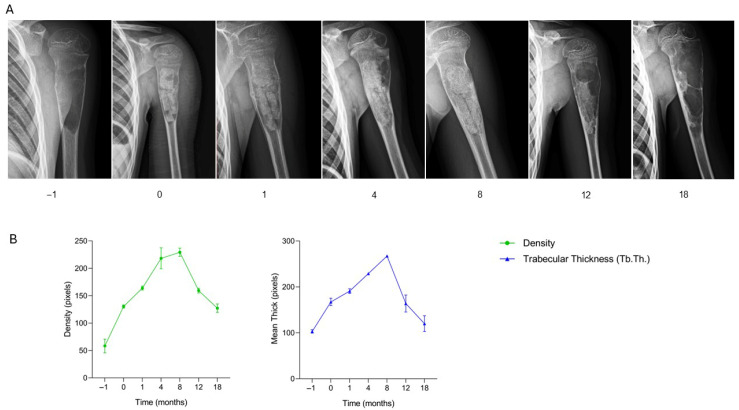
Radiographic follow-up after SmartBone^®^ grafting in a pediatric humeral bone cyst. (**A**) Serial radiographs show initial graft integration and remodeling (0–8 months), followed by lesion recurrence at 12–18 months. (**B**) Quantitative analysis of bone density and trabecular thickness (Tb.Th.) demonstrates a progressive increase until 4–8 months, consistent with clinical and radiographic improvement, followed by a decline from 12 months onward, coinciding with lesion recurrence.

**Figure 2:**
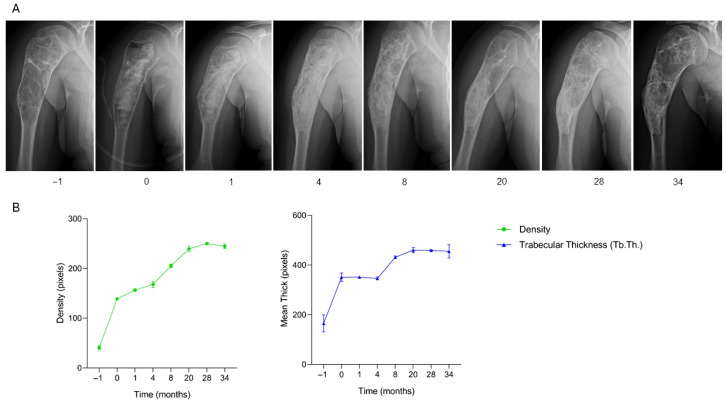
Radiographic follow-up after SmartBone^®^ grafting in a pediatric humeral bone cyst. (**A**) Serial radiographs show progressive graft integration and remodeling, with restoration of cortical continuity and no recurrence up to 34 months. (**B**) Quantitative analysis of bone density and trabecular thickness (Tb.Th.) demonstrates a gradual and continuous increase, reaching stable high levels by 34 months, consistent with sustained osteointegration and long-term functional recovery.

A 9-year-old male patient with a pathological fracture involving a bone cyst in the proximal left humerus underwent surgical curettage of the lesion and defect filling using two SmartBone^®^ sticks (35 × 4 × 3 mm) and one intraoperatively shaped SmartBone^®^ block (15 × 30 × 50 mm). Postoperative clinical assessment, recorded in the anonymized CRF, documented an initially favorable course, with progressive recovery of shoulder mobility and absence of pain. Radiographs at one month revealed early periosteal reaction along the cortical margins, indicating initial bone regeneration, consistent with the clinical improvement noted in the CRF. By four months, imaging confirmed progressive graft osteointegration and resolution of periosteal changes, correlating with continued functional recovery. At eight months, the grafts were well integrated, showing advanced remodeling and partial biomaterial resorption, paralleling the absence of symptoms and restored mobility reported in the CRF. However, at twelve months, radiographs identified a new lytic lesion in the proximal humerus, and at eighteen months, recurrence was confirmed, necessitating a second surgical intervention with lesion curettage, removal of previously implanted biomaterials, and filling with bank bone graft. Radiological findings (Figure 1A) thus closely corresponded with the clinical trajectory recorded in the CRF, illustrating the initial osteointegration, progressive remodeling, and eventual recurrence. Quantitative analysis (Figure 2B) reveals an early and consistent increase in bone density and trabecular thickness (Tb.Th.) up to 8 months, aligning with clinical and radiographic signs of graft osteointegration. Bone density peaked around 4–8 months before declining, coinciding with the onset of recurrence. Similarly, Tb.Th. followed a similar trend, confirming the temporary success of the treatment and later structural compromise due to lesion recurrence. This case demonstrates that SmartBone^®^ initially facilitated effective graft osteointegration, trabecular remodeling, and functional recovery of the proximal humerus. Nonetheless, the subsequent lesion recurrence highlights the importance of long-term radiographic surveillance and the potential need for secondary surgical intervention in pediatric patients with bone cysts complicated by pathological fractures.

##### Case ONCO-003

The patient, an 11-year-old boy diagnosed with a lytic bone cyst located in the proximal right humerus and complicated by a pathological fracture, underwent surgical treatment consisting of curettage of the lesion followed by filling of the bone defect with two SmartBone^®^ ORTHO blocks (dimensions 15 × 30 × 50 mm) combined with synthetic bioglass BoneAlive. Clinical data recorded in the anonymized CRF indicated regular postoperative progression, with full, pain-free shoulder range of motion (ROM) already at one month. At two months, radiographs revealed early bone apposition, consistent with the continued absence of pain and normal function reported in the CRF. By four months, imaging confirmed clear osteointegration, accompanied by complete recovery of both shoulder and elbow ROM. At nine months, the integration and mineralization of the grafted site were even more evident, correlating with the stable, asymptomatic clinical status. During follow-up assessments at 20, 24, 28, and 34 months, the patient maintained full, pain-free shoulder mobility and returned to normal daily activities. Serial radiographs (Figure 2A) illustrate the progressive healing process in the proximal humerus: preoperative imaging showed significant cortical disruption due to the fracture, while early postoperative X-rays reflected bone apposition and the onset of graft integration. Subsequent imaging over the following months demonstrated continuous bone consolidation, increased radiographic density, and restoration of cortical continuity, resulting in a well-integrated, compact structure. No signs of recurrence or postoperative complications were observed, confirming the long-term osteointegration and functional success of the implanted biomaterials. Quantitative analysis (Figure 2B) shows a gradual and continuous increase in bone density throughout the follow-up period, accompanied by a steady rise in trabecular thickness (Tb.Th.). Both parameters reached stable, high levels by the end of the 34-month observation, supporting the radiographic and clinical evidence of successful graft osteointegration and long-term structural consolidation. This case demonstrates the long-term efficacy of SmartBone^®^ combined with BoneAlive in achieving durable osteointegration, progressive bone remodeling, and full functional recovery, with sustained structural stability and absence of recurrence over a 34-month follow-up period.

##### Case ONCO-006

**Figure 3:**
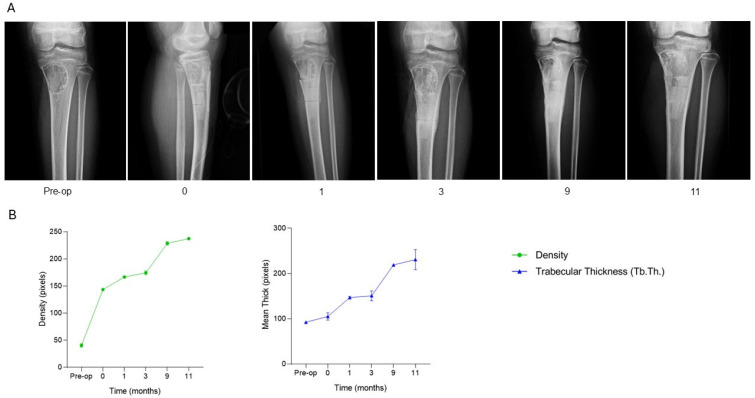
Radiographic follow-up after SmartBone^®^ grafting in a pediatric tibial non-ossifying fibroma. (**A**) Serial radiographs show progressive graft osteointegration, trabecular bridging, and increasing mineralization, with advanced cortical remodeling and full incorporation by 11 months. (**B**) Quantitative analysis of bone density and trabecular thickness (Tb.Th.) reveals a continuous increase throughout follow-up, confirming structural reinforcement and sustained functional recovery.

The patient, a 12-year-old female diagnosed with a pathological fracture on a non-ossifying fibroma located in the proximal left tibia, underwent surgical treatment consisting of curettage of the lesion followed by filling of the bone defect with one SmartBone^®^ block (dimensions 15 × 30 × 50 mm). At one month post-surgery, clinical data recorded in the anonymized CRF indicated regular progression, with good mobility and absence of pain. By three months, radiographs revealed bone apposition and cortical remodeling, consistent with maintained clinical stability. At nine months, osteointegration and mineralization of the implanted biomaterial were clear, demonstrating continuous structural evolution. Follow-up was completed at eleven months, during which the patient remained asymptomatic with full, pain-free range of motion, resuming normal daily and physical activities without limitations. Serial radiographs (Figure 3A) illustrate the healing trajectory of the proximal tibia. Preoperative imaging (time −1) revealed the lesion and fracture line within the metaphyseal region. Immediately post-surgery (time 0), the SmartBone^®^ block was clearly visible within the defect. At one and three months, progressive graft integration was evident, with trabecular bridging and increasing radiodensity. By nine and eleven months, the bone structure appeared denser and more continuous, indicating advanced mineralization and full incorporation of the graft into the native bone. Supporting quantitative graphs (Figure 3B) demonstrate a continuous increase in both bone density and trabecular thickness (Tb.Th.) over the eleven-month period. Bone density increased progressively, reflecting ongoing mineralization, while trabecular thickness showed a steady rise, indicating consistent bone regeneration and structural reinforcement. This case demonstrates that SmartBone^®^ enabled effective osteointegration, progressive mineralization, and structural reinforcement of the proximal tibia, resulting in full functional recovery, sustained bone stability, and pain-free resumption of daily and physical activities over the 11-month follow-up.

##### Case ONCO-007

**Figure 4:**
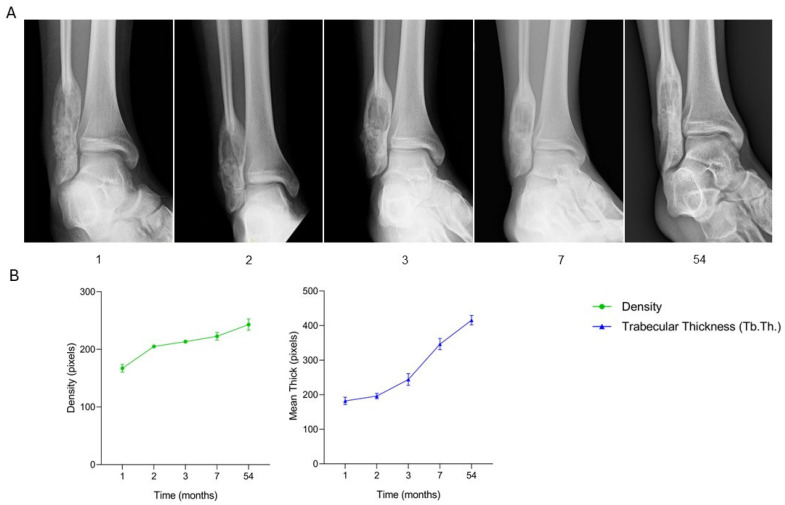
Radiographic follow-up after SmartBone^®^ grafting in a pediatric aneurysmal bone cyst. (**A**) Serial radiographs show progressive opacification and eventual homogeneous integration (1–54 months). (**B**) Quantitative analysis of bone density and trabecular thickness (Tb.Th.) demonstrates a continuous increase, consistent with clinical and radiographic improvement and long-term graft stability.

A 15-year-old male patient with an aneurysmal bone cyst in the distal right fibula (6 × 2.4 cm) underwent surgical curettage of the lesion and defect filling using two SmartBone^®^ blocks (15 × 30 × 60 mm). Postoperative clinical assessment, recorded in the anonymized CRF, documented a favorable course, with early recovery of ankle mobility. Radiographs at one month revealed a radiopaque area at the graft site, consistent with the presence of the biomaterial and early mineralization. The patient was allowed 50% weight-bearing with crutches and resumed swimming. At two months, radiographs showed progressive opacification, paralleled by full range of motion without pain and gradual discontinuation of crutches. By three months, imaging findings were stable, and the patient reported only occasional pain, while being able to resume physical activities including contact sports. At seven months, radiographs remained unchanged, with mild ankle discomfort but no pain on loading. From eleven to forty months, the patient remained asymptomatic, with full functional recovery and no limitations, progressively returning to sports including gym training. At fifty-four months, the patient was clinically well, and radiographs demonstrated complete and homogeneous integration of the graft with host bone. Radiological findings (Figure 4A) thus closely corresponded with the clinical trajectory recorded in the CRF, illustrating the initial mineralization, progressive remodeling, and eventual stable integration of the graft. Quantitative analysis (Figure 4B) reveals a continuous increase in bone density and trabecular thickness (Tb.Th.) throughout follow-up, confirming successful osteointegration and long-term graft stability. This case demonstrates that SmartBone^®^ blocks provided effective structural support and promoted progressive osteointegration in the treatment of an aneurysmal bone cyst of the distal fibula. At 54 months, the patient achieved complete clinical recovery and radiographic consolidation, with no evidence of recurrence, underscoring the long-term efficacy of this reconstructive strategy.

##### Case ONCO 032

**Figure 5:**
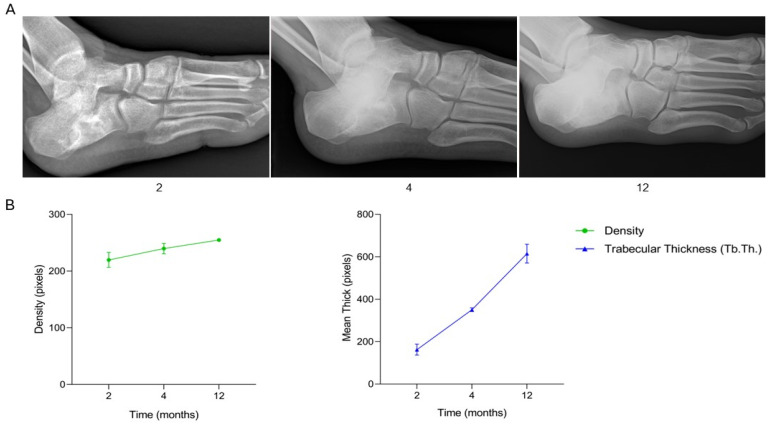
Radiographic follow-up after SmartBone^®^ grafting in a pediatric calcaneal bone cyst. (**A**) Serial radiographs show progressive graft osteointegration, reduction in radiolucency, and homogeneous bone consolidation by 12 months. (**B**) Quantitative analysis of bone density and trabecular thickness (Tb.Th.) demonstrates a steady increase throughout follow-up, confirming mineralization, structural maturation, and functional recovery.

The patient, a 16-year-old male diagnosed with a bone cyst in the left calcaneus, underwent surgical treatment consisting of curettage of the lesion followed by filling of the bone defect with one SmartBone^®^ ORTHO block (15 × 30 × 50 mm) in combination with a synthetic bone substitute (BoneAlive bioglass). At two months post-surgery, clinical data recorded in the anonymized CRF indicated progressive recovery, with gradual discontinuation of crutch use and only minimal wound dehiscence. By four months, the patient was ambulating unaided and reported no pain. At twelve months, the patient demonstrated full weight-bearing without limitations, consistent with radiographic evidence of ongoing graft integration. Serial radiographs (Figure 5A) illustrate the healing trajectory of the left calcaneus. Preoperative imaging (time −1) revealed the cystic lesion with a non-aggressive appearance. Immediately post-surgery (time 0), the SmartBone^®^ block and BoneAlive bioglass were clearly visible within the defect. At two months, residual radiolucency was noted, reflecting early phases of integration. By four months, the graft appeared more blended with surrounding bone, with less distinct margins, indicating ongoing remodeling. At twelve months, the treated area showed more homogeneous bone density, reduction in radiolucency, and radiographic signs consistent with successful osteointegration and bone consolidation. Supporting quantitative graphs (Figure 5B) demonstrate a progressive increase in both bone density and trabecular thickness (Tb.Th.) over the twelve-month period. Bone density increased steadily, reflecting ongoing mineralization, while trabecular thickness showed a continuous rise, indicating structural maturation of the newly formed bone. This case demonstrates that the combination of SmartBone^®^ ORTHO and BoneAlive bioglass promoted effective osteointegration, progressive bone remodeling, and structural maturation, resulting in full functional recovery, pain-free ambulation, and stable bone consolidation over the twelve-month follow-up.

##### Case ONCO-033

**Figure 6:**
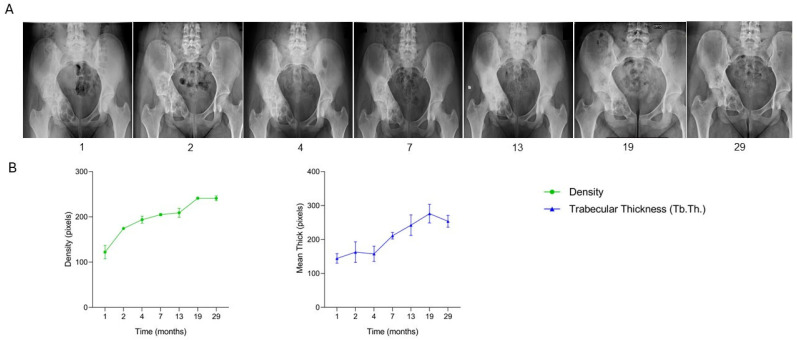
Radiographic follow-up after SmartBone^®^ grafting in a pediatric acetabular aneurysmal bone cyst. (**A**) Serial radiographs show progressive graft osteointegration, reduction in radiolucency, and increasing mineralization, with advanced consolidation and stable bone profile by 29 months. (**B**) Quantitative analysis demonstrates a steady increase in bone density, plateauing after 19 months, while trabecular thickness (Tb.Th.) showed a slight late decrease, consistent with physiological remodeling.

The patient, a 16-year-old female diagnosed with an aneurysmal bone cyst involving the articular surface of the acetabulum and the right iliopubic branch, underwent surgical treatment consisting of curettage of the lesion followed by filling of the bone defect with one SmartBone^®^ ORTHO block (15 × 30 × 60 mm) in combination with a synthetic bone substitute (BoneAlive bioglass). At two, four, and seven months post-surgery, clinical data recorded in the anonymized CRF indicated good local progression, with gradual recovery of function. By thirteen months, the patient exhibited full, pain-free hip ROM. At nineteen months, she had resumed sports activities without limitations, and follow-up at twenty-three and twenty-nine months demonstrated stable clinical status. Serial radiographs (Figure 6A) illustrate the healing trajectory over a 29-month period. Preoperative imaging (time −1) showed the cystic lesion affecting the acetabulum and iliopubic branch. Immediately post-surgery (time 0), the SmartBone^®^ block and BoneAlive bioglass were clearly visible within the defect. Between one and seven months, radiographs showed gradual graft integration with native bone, reduction in radiolucency, and early signs of mineralization. By thirteen months, the treated area appeared denser and more homogeneous, reflecting successful structural integration consistent with restored, pain-free hip mobility. At nineteen and twenty-nine months, imaging revealed further maturation and consolidation of the bone tissue, with increased radiographic density and a more regular profile, in line with the patient’s return to physical activity. At 29-month follow up an asymptomatic osteolytic area in the pubic ramus was noticed (out of the curetted and grafted zone) and treated with minimally invasive curettage and bone paste injection not affecting the previously grafted area. Supporting quantitative graphs (Figure 6B) demonstrated a steady increase in bone density over time, with a plateau between nineteen and twenty-nine months, indicating structural stability of the graft. Trabecular thickness (Tb.Th.) showed a slight decrease at twenty-nine months, consistent with physiological bone remodeling. Radiographic and quantitative data together confirm successful osteointegration of the composite SmartBone^®^ graft. These findings correlate with clinical recovery, marked by full joint functionality and pain-free resumption of sports activities. Final radiographs confirm the long-term success of the treatment. This case demonstrates that the composite SmartBone^®^ ORTHO and BoneAlive bioglass graft enabled successful long-term osteointegration, structural consolidation, and functional restoration of the acetabular and iliopubic regions, allowing full, pain-free hip mobility and safe return to sports over a 29-month follow-up period.

##### Case ONCO-034

**Figure 7:**
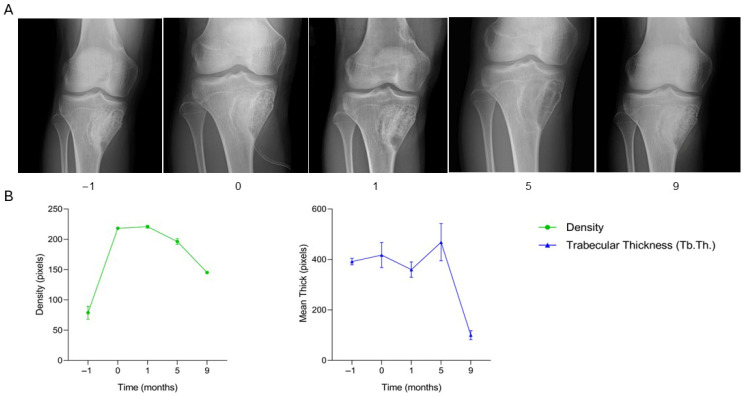
Radiographic follow-up after initial SmartBone^®^ grafting in a proximal tibial aneurysmal bone cyst. (**A**) Serial radiographs (−1 to 9 months) show initial graft integration and cortical healing after curettage and filling with granular SmartBone^®^, followed by progressive remodeling. At nine months, recurrence is evident with cortical discontinuity and reduced trabecular organization. (**B**) Quantitative analysis of bone density and trabecular thickness (Tb.Th.) shows an early increase after surgery, consistent with graft integration, followed by a marked decline at nine months corresponding to lesion recurrence.

**Figure 8:**
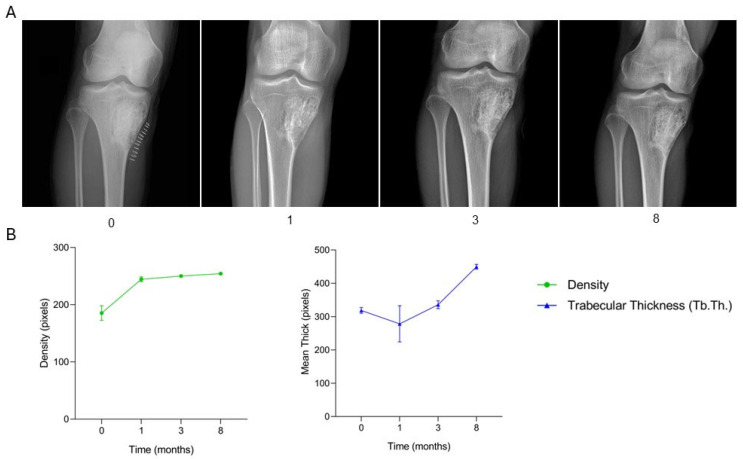
Radiographic follow-up after revision surgery with SmartBone^®^ grafting. (**A**) Serial radiographs (0 to 8 months) demonstrate good positioning of the SmartBone^®^ block, with progressive cortical reformation and trabecular organization, without signs of recurrence. (**B**) Quantitative analysis of bone density and trabecular thickness (Tb.Th.) reveals a steady increase after revision, indicating successful osteointegration and structural bone regeneration.

The patient, a 17-year-old male diagnosed with an aneurysmal bone cyst located in the proximal right tibia, underwent surgical treatment consisting of curettage of the lesion followed by filling of the bone defect with granular SmartBone^®^ (5.5 g, 2–4 mm) and coverage with Spongostan^®^, a sterile, absorbable hemostatic gelatin of porcine origin. At two months post-surgery, clinical data recorded in the anonymized CRF indicated progressive cortical healing, absence of symptoms, and gradual return to sports activity. By five months, the patient remained asymptomatic, although a minor cortical discontinuity persisted. At nine months, the patient reported exertional pain, and radiographs suggested a local recurrence. A revision procedure was performed at fourteen months, involving removal of the recurrent cyst and filling of the bone defect with a SmartBone^®^ block (15 × 30 × 50 mm). One month post-revision, the patient was pain-free and exhibited full range of motion. By three months, clinical assessment confirmed resumption of sports activities, and radiographs showed progressive graft integration without signs of recurrence. At eight months, the patient remained asymptomatic, engaged in regular physical activity, and demonstrated normal limb function. Serial radiographs (Figure 7A and Figure 8A) illustrate the healing trajectory before and after revision surgery. Preoperative imaging showed a large lytic area with medial cortical disruption and trabecular rarefaction. Post-initial surgery, the defect was filled with granular SmartBone^®^, showing good compaction, early integration, and partial cortical reconstruction. At nine months, recurrence was evident with a new radiolucent area, decreased trabecular organization, and clinical pain. Following the revision, radiographs showed the SmartBone^®^ block well-positioned within the defect, with progressive cortical reformation and trabecular organization up to eight months. Supporting quantitative graphs (Figure 7B and Figure 8B) show a progressive increase in bone density and trabecular thickness (Tb.Th.) after the initial surgery, with a marked decline at nine months corresponding to recurrence. Post-revision, both parameters steadily increased and stabilized, indicating effective osteointegration and structural bone regeneration. This case demonstrates two distinct phases: an initial positive response with graft integration, followed by recurrence and structural compromise, and finally successful restoration of bone architecture and function after revision surgery. This case illustrates that SmartBone^®^ can achieve initial osteointegration and structural regeneration, while highlighting that recurrence may occur and that revision surgery can restore graft stability, bone architecture, and full functional recovery.

##### Case ONCO-035

**Figure 9:**
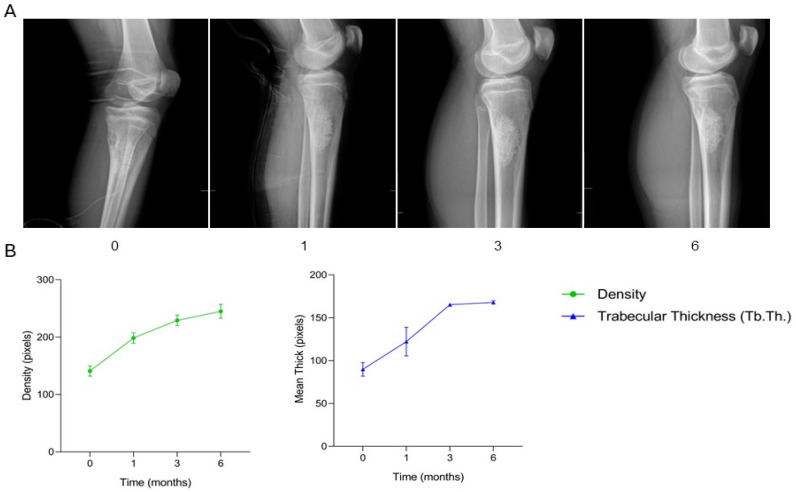
Radiographic follow-up after SmartBone^®^ grafting in a pediatric tibial non-ossifying fibroma. (**A**) Serial radiographs show early graft incorporation at one month, progressive trabecular organization at 3 months, and complete osteointegration with native bone by 6 months. (**B**) Quantitative analysis demonstrates an initial decrease in bone density followed by a steady increase, together with progressive rise in trabecular thickness (Tb.Th.), confirming successful graft remodeling and structural reinforcement consistent with full functional recovery.

The patient, a 15-year-old male diagnosed with a non-ossifying fibroma located in the proximal metaphysis of the left tibia, underwent surgical treatment consisting of curettage of the lesion followed by filling of the bone defect with two granular SmartBone^®^ grafts (5.5 g, 2–4 mm). At one month post-surgery, clinical data recorded in the anonymized CRF indicated progressive recovery, with the patient able to walk without assistance and achieve full weight-bearing. By three months, radiographs revealed early signs of osteointegration, consistent with maintained clinical stability. At six months, osteointegration and trabecular organization were clear, and the patient had resumed normal daily activities without limitations. Serial radiographs (Figure 9A) illustrate the healing trajectory of the proximal tibia. Preoperative imaging (time −1) revealed the lesion within the metaphyseal region. Immediately post-surgery (time 0), the granular graft material was clearly visible within the defect, with homogeneous radiodensity and well-defined margins. At one month, early osteointegration was observed, with a slight decrease in graft density reflecting physiological resorption and initial bone remodeling. By three months, increased radiodensity and early trabecular organization indicated ongoing incorporation of the graft. At six months, the graft appeared fully integrated, with radiodensity similar to adjacent native bone and a well-structured trabecular pattern, corresponding to the patient’s unrestricted ambulation and return to normal activities. Supporting quantitative graphs (Figure 9B) show a physiological decrease in bone density during the initial postoperative period, followed by a steady increase up to six months, reflecting successful graft integration. Trabecular thickness (Tb.Th.) progressively increased over the same period, reaching maximal values at six months. Radiographic and quantitative analyses confirm excellent osteointegration and structural reinforcement of the SmartBone^®^ grafts in the proximal tibia, in line with the clinical course of full recovery. This case demonstrates that granular SmartBone^®^ grafts enabled effective osteointegration, progressive trabecular organization, and structural reinforcement of the proximal tibia, resulting in full functional recovery and return to normal daily activities within six months.

##### Case ONCO-036

**Figure 10:**
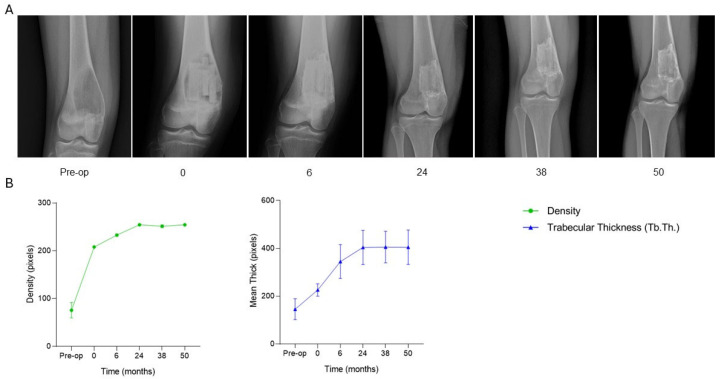
Radiographic follow-up after SmartBone^®^ grafting in a distal femoral aneurysmal bone cyst. (**A**) Serial radiographs show correct positioning of two SmartBone^®^ blocks and one wedge immediately post-surgery, early graft integration at 6 months, progressive incorporation at 24 months, and complete structural regeneration with homogeneous bone morphology at 38 and 50 months. (**B**) Quantitative analysis reveals a rapid increase in bone density within the first months, followed by long-term stabilization, together with a marked rise in trabecular thickness (Tb.Th.) up to 24 months and maintenance, thereafter, confirming durable osteointegration and structural maturation.

The patient, a 17-year-old female diagnosed with an aneurysmal bone cyst in the distal right femur, underwent surgical treatment consisting of curettage of the lesion followed by filling of the bone defect with two SmartBone^®^ blocks (15 × 30 × 50 mm) and one SmartBone^®^ wedge (40 × 25 × 14 mm). At two months post-surgery, clinical data recorded in the anonymized CRF indicated full knee flexion, absence of pain, and independent ambulation without assistive devices. Early radiographic signs of graft integration were also observed. By six months, the patient remained asymptomatic and had gradually resumed normal daily activities, with continued graft integration. At 26 months, radiographs confirmed optimal incorporation of the grafts into the host bone. By 38 months, the patient had regained full range of motion, remained pain-free, and the grafts were fully integrated. Serial radiographs (Figure 10A) illustrate the healing trajectory of the distal femur. Preoperative imaging (time −1) showed a large osteolytic lesion in the distal diaphysis, consistent with reported pain. Immediate postoperative imaging (time 0) revealed correct positioning of the two SmartBone^®^ blocks and wedge, sharply delineated from the surrounding bone. At six months, early integration was evident, with progressive blurring of graft margins. By 24 months, the treated area appeared increasingly homogeneous, reflecting advanced structural incorporation. Radiographs at 38 and 50 months confirmed complete regeneration, with morphology and density closely resembling the surrounding bone tissue. Clinically, the patient remained asymptomatic, with full recovery of joint mobility and return to normal daily activities without limitations. Supporting quantitative graphs (Figure 10B) demonstrate a rapid increase in bone density immediately post-surgery, followed by continued rise over the first six months and stabilization during long-term follow-up, reflecting progressive mineralization and successful graft integration. Trabecular thickness (Tb.Th.) increased markedly over the first 24 months and plateaued through 50 months, supporting structural maturation and stable osteointegration. This case demonstrates that SmartBone^®^ blocks and wedge can achieve progressive and complete osteointegration, restore bone structure, and ensure long-term functional recovery in the distal femur, with stable graft incorporation, full joint mobility, and pain-free return to daily activities maintained over more than four years of follow-up.

##### Case ONCO-040

**Figure 11:**
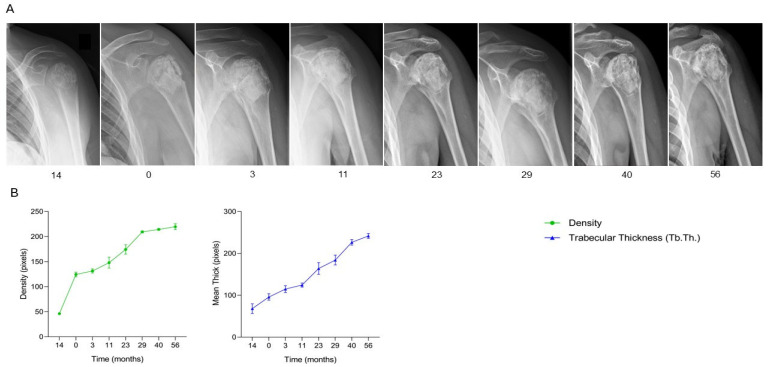
Radiographic follow-up after SmartBone^®^ grafting in a proximal humeral chondroblastoma. (**A**) Serial radiographs after revision surgery show progressive graft incorporation and remodeling, with complete structural integration and restoration of bone morphology up to 56 months. (**B**) Quantitative analysis demonstrates a steady increase in bone density and trabecular thickness (Tb.Th.) during the first months post-revision, followed by long-term stabilization, correlating with full functional recovery and sustained absence of recurrence.

The patient, a 16-year-old male diagnosed with a chondroblastoma in the proximal left humerus, underwent initial surgical treatment consisting of curettage of the lesion followed by filling of the bone defect with two boxes (5.5 g) of SmartBone^®^ granules (2–4 mm) and one SmartBone^®^ block (15 × 30 × 60 mm). The immediate postoperative course was uneventful, with progressive recovery of shoulder mobility. At 13 months post-surgery, the patient presented with local pain and restricted range of motion, and radiographs revealed a periarticular inflammatory reaction. Biopsy confirmed local recurrence of the tumor. A second surgical procedure was performed, involving removal of the previously implanted biomaterials, repeat curettage, and reconstruction of the defect with the same SmartBone^®^ combination. At three months post-revision, clinical data recorded in the anonymized CRF indicated partial recovery of shoulder motion, with active elevation of 80° and abduction of 45°, and pain only during extreme passive movements. Radiographs documented favorable graft integration. By eight months, the patient showed substantial clinical improvement, with pain well-controlled and shoulder elevation and abduction reaching 160° and 150°, respectively. At 17 months, full range of motion was restored, and radiographs confirmed stable and advanced osteointegration of the grafts. Long-term follow-up at 29, 34, 40, 48, and 56 months demonstrated that the patient remained asymptomatic, with preserved joint function and no radiographic evidence of recurrence. Serial radiographs (Figure 11A) illustrate progressive osteointegration and remodeling of the grafts, with complete structural incorporation following the second reconstruction. Supporting quantitative graphs (Figure 11B) show a consistent increase in bone density and trabecular thickness (Tb.Th.) during the early months post-revision, followed by stabilization over time, correlating with clinical findings. This case demonstrates that SmartBone^®^ granules and block can achieve durable and effective osteointegration even in the context of tumor recurrence, supporting complete restoration of bone structure, long-term stability, and full functional recovery of the shoulder over more than four years of follow-up.

##### Case ONCO-041

**Figure 12:**
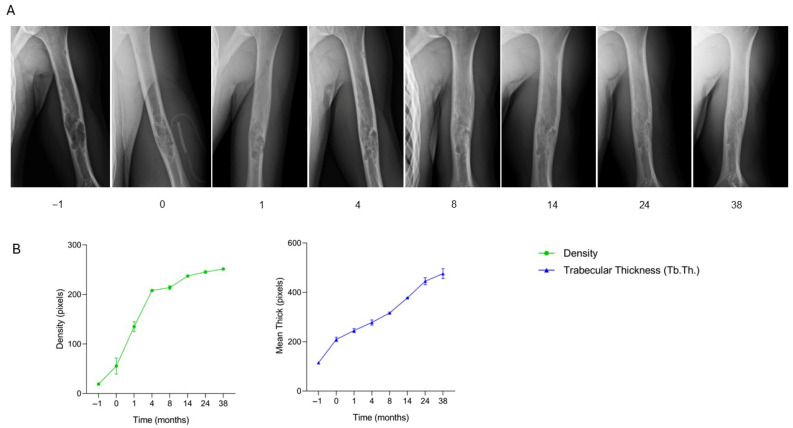
Radiographic follow-up after SmartBone^®^ grafting in a distal humeral bone cyst. (**A**) Serial radiographs show correct positioning of two SmartBone^®^ blocks immediately post-surgery, early integration and increased radiopacity at 1 and 4 months, progressive bone filling and cortical formation at 8 months, and complete structural incorporation with cortical continuity at 14, 24, and 38 months. (**B**) Quantitative analysis demonstrates a continuous rise in bone density and trabecular thickness (Tb.Th.) throughout follow-up, confirming progressive mineralization, substantial structural regeneration, and stable osteointegration consistent with full functional recovery.

The patient, a 16-year-old male diagnosed with a bone cyst in the distal left humerus, underwent surgical treatment consisting of curettage of the lesion followed by filling of the bone defect with two SmartBone^®^ blocks (14 × 12 × 12 mm). At one month post-surgery, clinical data recorded in the anonymized CRF indicated full shoulder range of motion without peripheral neurovascular deficits. Early radiographs showed a slight increase in bone radiodensity, consistent with initial graft integration. By four months, radiographs demonstrated ongoing osteointegration of the implanted biomaterial. At eight months, the patient was pain-free with complete range of motion, and imaging revealed substantial bone filling and cortical formation. Follow-up at 14, 24, and 38 months confirmed full graft integration, excellent bone regeneration, and restoration of cortical continuity. Clinically, the patient maintained full joint mobility and had resumed sporting activities without limitations. Serial radiographs (Figure 12A) illustrate the healing progression in the distal humerus. Preoperative imaging (time −1) showed the bone cyst within the metaphyseal region. Immediate postoperative radiographs (time 0) clearly visualized the SmartBone^®^ blocks within the defect. At one and four months, progressive graft integration and increasing radiopacity were observed. By eight months, further bone filling and cortical formation were evident. Radiographs at 14, 24, and 38 months confirmed complete mineralization of the graft and structural incorporation into the native bone. Supporting quantitative graphs (Figure 12B) demonstrate a marked and continuous increase in both bone density and trabecular thickness (Tb.Th.) throughout the 38-month follow-up. Bone density increased steadily, reflecting progressive mineralization, while trabecular thickness showed continuous growth, indicating substantial and sustained structural bone regeneration. This case demonstrates that SmartBone^®^ blocks can achieve progressive and complete osteointegration in the distal humerus, restoring bone structure and function. Radiographic and quantitative data correlate with the clinical course, showing full pain-free recovery, restoration of shoulder mobility, and return to daily and athletic activities without limitations.

##### Case ONCO-044

**Figure 13:**
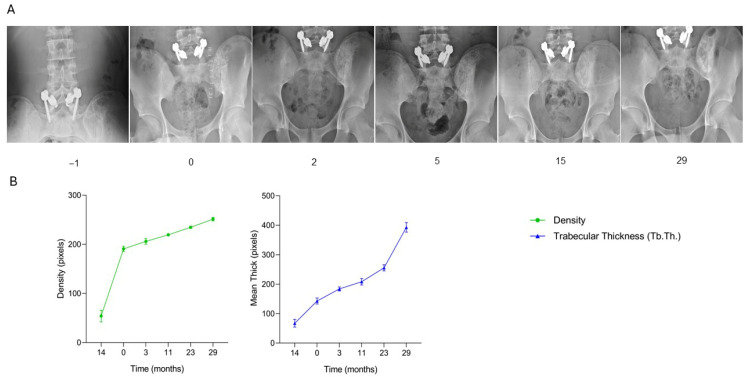
Radiographic follow-up after SmartBone^®^ scaffold grafting in an iliac wing bone cyst. (**A**) Serial radiographs show early trabecular reorganization at 2 and 5 months, progressive remodeling and osteointegration at 15 months, and complete structural incorporation at 29 months, with the treated area indistinguishable from native bone. (**B**) Quantitative analysis demonstrates a continuous increase in bone density and trabecular thickness (Tb.Th.) over time, confirming ongoing mineralization, trabecular maturation, and stable long-term osteointegration.

The patient, a 16-year-old male diagnosed with a bone cyst measuring 6.5 × 2.5 × 5 cm in the left iliac wing, underwent surgical treatment consisting of curettage of the lesion followed by filling of the bone defect with two SmartBone^®^ rods (35 × 4 × 3 mm) and one morcellated SmartBone^®^ scaffold, shaped intraoperatively to adapt to the residual cavity. At one and two months post-surgery, clinical data recorded in the anonymized CRF indicated regular progression, with good wound healing, absence of secretion, and radiographs free of complications. By five months, radiological evaluation demonstrated evident reparative activity and early graft integration. Follow-up at 15 and 16 months revealed progressive consolidation of the bone defect, with radiographs confirming further remodeling and advanced osteointegration. At 29 months, the treated area appeared fully consolidated and stable, with no signs of recurrence or local complications. Clinically, the patient remained asymptomatic, with no functional limitations and an excellent recovery. Serial radiographs (Figure 13A) illustrate the healing trajectory of the left iliac wing. Preoperative imaging (time −1) showed a large cystic lesion with marked thinning of the iliac profile. Immediate postoperative radiographs (time 0) clearly visualized the SmartBone^®^ rods and morcellated scaffold, well-positioned within the defect and distinct from the surrounding bone. At five months, early trabecular reorganization was evident, with a more homogeneous structure. By 15 and 16 months, further remodeling occurred, with blurring of the margins between graft and host bone, consistent with progressive osteointegration. Radiographs at 29 months confirmed complete remodeling, with the treated area indistinguishable from native bone. Supporting quantitative graphs (Figure 13B) demonstrate a continuous increase in both bone density and trabecular thickness (Tb.Th.) throughout the 29-month follow-up, reflecting ongoing mineralization and maturation of the trabecular network. This case demonstrates that SmartBone^®^ rods combined with a morcellated scaffold can achieve progressive and complete osteointegration in large iliac cystic defects, restoring bone structure and function. Radiographic, quantitative, and clinical findings are fully aligned, confirming stable graft incorporation, absence of recurrence, and excellent functional recovery.

### 2.2. Molecular Interpretation of Radiographic Remodeling

The progressive radiographic increase in bone density and trabecular thickness (Tb.Th.) observed in our cohort reflects the underlying cellular and molecular processes of bone regeneration and SmartBone^®^ remodeling. These patterns are consistent with previously documented histological and in vitro findings demonstrating the capacity of SmartBone^®^ to sustain cell adhesion, proliferation, and osteogenic differentiation [17,18].

In the early postoperative period, the implanted graft rapidly absorbs blood, initiating a local micro-coagulation cascade. This process releases a wide range of growth factors such as PDGF, VEGF, and TGF-β, which stimulate the recruitment of mesenchymal stromal cells (MSCs) and endothelial progenitors from the surrounding tissue [19]. These MSCs adhere to the RGD-rich gelatin component of SmartBone^®^ through integrin-mediated binding (mainly α5β1 and αvβ3), activating focal adhesion kinase (FAK) and downstream ERK and PI3K/Akt signaling pathways. Such activation triggers cytoskeletal rearrangement and promotes the expression of osteogenic transcription factors like RUNX2 and Osterix, leading to commitment toward the osteoblastic lineage [20,21].

The open, interconnected porosity of SmartBone^®^ (mean pore size ≈ 250 µm) ensures rapid vascular invasion and nutrient exchange, supporting the coupling of angiogenesis and osteogenesis essential for graft integration. As osteoblasts deposit new matrix and the PLCL copolymer gradually hydrolyzes, the mineral phase becomes accessible for cellular resorption and remodeling. Over the following months, radiographic density and Tb.Th. increase proportionally to the deposition and maturation of mineralized tissue, culminating in the formation of organized lamellar bone indistinguishable from the surrounding native bone.

Thus, the radiological evolution observed during follow-up mirrors the underlying molecular and cellular cascade of bone repair, confirming that SmartBone^®^ acts not as a passive filler but as a bioactive scaffold guiding osteogenesis and vascular integration.

## 3. Discussion

The management of bone defects in pediatric patients, resulting from oncologic resections or the treatment of pseudotumoral and benign neoplastic lesions, still represents one of the most challenging issues in orthopedic oncology [22]. The primary goal remains the preservation of joint function and weight-bearing capacity, while minimizing the risk of recurrence, complications, and interference with skeletal growth. In this context, the search for bone substitute materials capable of combining adequate biomechanical properties, effective osteoconduction, and the potential for physiological remodeling has progressively gained relevance [23].

In the present study, we reported the clinical and radiological outcomes of a series of 12 pediatric patients treated with SmartBone^®^ ORTHO, a xeno-hybrid graft composed of deproteinized bovine bone matrix enriched with collagen fragments and synthetic biopolymers. The analysis documented a success rate of 75% at first surgery and 100%, including post-relapses revision surgeries, with good osteointegration in most cases and local recurrence only in three patients. These recurrences involved a simple bone cyst, an aneurysmal bone cyst, and a chondroblastoma, lesions which are known from the literature to carry an intrinsic risk of recurrence even when treated with aggressive curettage and filling using other materials [24,25]: the failure was proven not attributable to the biomaterial itself, but to the biological behavior of the lesions, as two of these were treated in a second surgery again with SmartBone^®^, and results were successful in the end, also for the third case treated with allograft.

From a radiological perspective, quantitative analysis using ImageJ and the BoneJ2 plugin enabled us to objectively assess the process of mineralization and bone remodeling. In all cases with favorable outcomes, a progressive increase in bone density and trabecular thickness was observed, with values stabilizing between 6 and 12 months and reaching levels comparable to healthy bone. This finding confirms the osteoconductive capacity of the material and its potential for complete replacement by new bone tissue, in line with what has been reported in experimental studies on SmartBone^®^ in adult clinical series [26,27].

Another relevant aspect concerns the biological tolerance and the absence of infectious or inflammatory complications during the entire follow-up up to the date of this paper. None of the patients experienced rejection phenomena, material-related infections, or significant delays in healing. This further confirms known literature evidence supporting both the safety and efficacy of the SmartBone^®^ technology and the validity of its xeno-hybrid composition, which preserves a porous and interconnected matrix suitable for cellular and vascular ingrowth while providing high mechanical performance and sparking complete remodeling over healing timeframe.

When compared with traditional strategies, additional advantages can be highlighted. Autologous bone grafting, although considered the gold standard for osteoinductive capacity and integration, is limited in pediatric patients due to poor availability and the risk of donor-site morbidity [28]. Allografts provide a larger reserve but present biological limitations, risks of resorption, fractures, and infectious complications [29]. Conventional xenografts often show limitations in mechanical strength and long-term remodeling. In this scenario, SmartBone^®^ appears to offer an intermediate solution, with biomechanical characteristics superior to traditional bovine grafts and greater biological integration than allografts, as confirmed by the results here presented.

At the molecular and cellular level, the integration and remodeling of SmartBone^®^ can be explained by its composite architecture and biofunctional surface chemistry. The graft is composed of a bovine-derived mineral matrix coated with poly(L-lactide-co-ε-caprolactone) (PLCL) and gelatin-derived collagen fragments containing Arg-Gly-Asp (RGD) sequences. These RGD motifs provide specific binding sites for integrin receptors (mainly α5β1 and αvβ3) expressed on mesenchymal stromal cells, osteoblasts, and endothelial cells. Their engagement activates intracellular pathways—particularly FAK, ERK1/2, and PI3K/Akt—leading to cytoskeletal organization and upregulation of osteogenic transcription factors such as RUNX2, ALP, and osteocalcin [20,21]. This signaling cascade promotes osteogenic differentiation, matrix deposition, and the establishment of early bone micro-niches within the graft.

Simultaneously, the open and interconnected porosity of SmartBone^®^ facilitates rapid vascular ingrowth, allowing nutrient diffusion and coupling between angiogenesis and osteogenesis. The gradual hydrolysis of PLCL releases biocompatible degradation products and progressively exposes the underlying mineral phase, which maintains a carbonate-substituted hydroxyapatite crystal structure similar to that of young human bone [30,31]. This phase is readily remodeled by osteoclasts and osteoblasts through physiological bone turnover mechanisms, enabling full substitution by newly formed lamellar bone.

These molecular events are consistent with the radiological findings observed in our cohort, where increased density and trabecular thickness reflected progressive mineral deposition and structural maturation. The correspondence between molecular pathways and our imaging results supports the dual behavior of SmartBone^®^ as both osteoconductive and osteoinductive, providing a structural scaffold while actively stimulating bone formation through integrin-mediated signaling and growth factor activation.

From a clinical perspective, findings of the present study showed that the biomaterial was effective not only in benign lesions but also in a case of osteosarcoma, where the post-resection defect was stabilized with a custom-made graft combined with internal fixation. In this patient, progressive radiological integration was observed up to 18 months, with preserved joint function and mechanical stability, demonstrating the versatility of the material even in complex oncologic contexts.

Nevertheless, this study has some limitations also connected to the specific investigated population, i.e., the rare sub-set of pediatric oncology patients: although representative of different lesion types and anatomical sites, the sample size was small and did not allow for comparative statistical analyses. The retrospective design might introduce potential selection bias, and the follow-up, although extended beyond three years, is still weak in statistically assessing the long-term impact of the biomaterial on skeletal growth, an especially critical issue in pediatric patients. Moreover, the heterogeneity of diagnoses limits the possibility of drawing definitive conclusions for specific subgroups of pathologies.

Despite these limitations, our findings contribute to a growing body of evidence supporting the use of xeno-hybrid biomaterials in such complex reconstructive surgery. Similar results have been reported in adult series in trauma, prosthetic, and oncologic surgery, suggesting a good level of reliability and predictability of this material [24,27]. Our experience extends this scenario to the pediatric field, paving the way for future prospective multicenter studies with larger samples and standardized protocols.

Looking ahead, further investigations should focus on: (1) the remodeling dynamics of the biomaterial in relation to skeletal growth, and (2) its long-term performance in patients requiring subsequent revision surgeries.

## 4. Materials and Methods

### 4.1. Clinical Data

The present study follows the STrengthening the Reporting of Observational Studies in Epidemiology (STROBE) guidelines. All procedures adhered to the ethical principles set forth in the Declaration of Helsinki (2013) and complied with good clinical practice (GCP). The study protocol was approved by the United Ethical Committee of the “C.T.O. Centro Traumatologico Ortopedico” in Turin, Italy (approval nr. 0004336 dated 15 January 2018, protocol nr. CS2/526), covering the “Regina Margherita” hospital where all patients were treated. Written informed consent was obtained from all patients’ legal parental authority, ensuring they understood the study objectives and authorized the use of their medical data for research purposes.

### 4.2. Study Design, Patients’ Selection, and Endpoints

A retrospective analysis of a prospectively maintained database of 12 pediatric patients (9 males, 3 females; age range 8–17 years at surgery; mean age ~14.6 years) who underwent surgery for musculoskeletal tumors or benign bone lesions between December 2016 and December 2019 was conducted. In selected cases, the surgical team performed bone defect reconstruction using SmartBone^®^ ORTHO, sometimes in combination with adjuncts (e.g., bioglass BoneAlive, Spongostan^®^ (Ferrosan Medical Devices, Søborg, Denmark)) and, when indicated, internal fixation (e.g., plate and screws in the osteosarcoma case). Informed consent was obtained from parents or legal guardians in accordance with Good Clinical Practice (GCP), including consent for anonymous use of clinical data for research.

The use of SmartBone^®^ ORTHO was considered the most suitable option when alternative grafts were limited or de facto unavailable, based on a patient-specific assessment (anatomical site, lesion biology, mechanical requirements) and the surgical team’s experience. The study draws upon clinical data and radiological images collected pre-operatively, immediately post-operatively, and during scheduled follow-up.

A total of 12 pediatric patients (9 males, 3 females; mean age 14.6 years, range 8–17) were included in this series. The patients presented with different benign bone lesions localized in the long bones, pelvis, and calcaneus. SmartBone^®^ ORTHO was employed after intralesional curettage and, when necessary, associated with other biomaterials or fixation systems. Clinical and radiological follow-up confirmed good graft integration in most cases, with revision surgery required in selected patients due to recurrence or surgery related complications, both quite common in these types of complex cases.

The distribution of lesions was as follows:

- Proximal humerus: 2 bone cysts (with pathological fracture), 1 chondroblastoma;
- Distal humerus: 1 simple bone cyst;
- Distal femur: 1 aneurysmal bone cyst;
- Proximal tibia: 1 non-ossifying fibroma, 2 aneurysmal bone cysts;
- Distal fibula: 1 aneurysmal bone cyst;
- Calcaneus: 1 simple bone cyst;
- Pubic ramus/acetabulum: 1 aneurysmal bone cyst;
- Iliac wing: 1 simple bone cyst.

Diagnoses included:

- Simple bone cysts: 4 cases (proximal humerus, distal humerus, calcaneus, iliac wing);
- Aneurysmal bone cysts: 4 cases (proximal tibia, distal femur, distal fibula, pubic ramus/acetabulum);
- Non-ossifying fibroma: 1 case (proximal tibia);
- Chondroblastoma: 1 case (proximal humerus);
- Other benign lesion: 2 cases (bone cysts with pathological fracture).

Revision surgery was required in 3 cases:

- ONCO-001: recurrence of a bone cyst in the proximal humerus treated with curettage and allograft bone paste.
- ONCO-034: recurrence of an aneurysmal bone cyst in the proximal tibia, treated with repeat curettage and SmartBone^®^ block.
- ONCO-040: recurrence of a chondroblastoma in the proximal humerus, treated with repeat curettage and SmartBone^®^.

All other patients achieved stable graft integration and functional recovery without the need for further surgical procedures.

The inclusion criteria are:

- Pediatric patients (<18 years) requiring curettage and defect reconstruction with SmartBone^®^ ORTHO
- Open growth plates at the time of surgery
- Availability of complete clinical and radiological follow-up

The exclusion criteria are:

- Age ≥18 years
- Severe systemic comorbidities (e.g., metabolic bone disease, dialysis-dependent renal disease, uncontrolled diabetes) or active extra-skeletal malignancies

Primary endpoints were radiographic graft osteointegration and functional recovery. Secondary endpoints included the need for revision surgery due to recurrence or complications (e.g., pseudoarthrosis, infection, implant removal). Revision procedures occurred in three cases (one simple bone cyst recurrence, one chondroblastoma recurrence, one aneurysmal bone cyst recurrence). The remaining patients achieved stable graft incorporation without further surgery.

Demographic and biomechanical data from patients included in the study are presented in the following table (Table 1).

### 4.3. Clinical Monitoring via Case Report Forms

The qualitative evaluation of patients’ clinical status was performed at three distinct time points: before the surgical procedure, immediately after the intervention, and during the scheduled follow-up visits, to accurately monitor clinical evolution over time. This assessment was conducted through the analysis of anonymized Case Report Forms (CRFs) completed by the treating physicians according to standardized protocols. The CRFs allowed for the systematic collection of detailed clinical information, including patient-reported symptoms, the functionality and condition of the anatomical site involved, any post-operative complications, and the progress of recovery. The use of standardized forms ensured uniformity in data collection across different operators, enhancing the comparability of results. All procedures for data collection and management were carried out in compliance with current regulations on personal data protection, ensuring the complete confidentiality of patients.

### 4.4. Radiological Data Analysis

Radiographic images of the patients were analyzed using ImageJ software (version 2.9.0) (National Institutes of Health, Bethesda, MD, USA) [32]. For each patient, the analysis included preoperative radiographs, acquired at the time of diagnosis of the bone lesion; immediate postoperative images, taken after surgical resection and implantation of the SmartBone^®^ ORTHO graft; and follow-up radiographs, obtained at regular intervals to monitor the progression of the bone regeneration process.

The analysis focused on the bone area of interest (Region of Interest, ROI), with the aim of evaluating the changes in bone density [32], estimated by the mean gray value within the ROI, and the changes in trabecular thickness (Tb.Th.), quantified as the average distance between adjacent trabeculae within the ROI [33].

#### 4.4.1. Radiographic Image Preparation

- Region of Interest (ROI) Selection

For each image, a Region of Interest (ROI) was manually defined using the “Freehand Selection” tool in ImageJ. The selected ROI encompassed the area of the bone lesion, the surgical resection, and the scaffold implantation.

- Application of Gaussian Blur Filter

To reduce noise and improve image quality, a Gaussian Blur filter was applied within the selected ROI, using a Sigma value between 1 and 2 pixels. This procedure allowed the preservation of trabecular structures while minimizing background irregularities.

- Threshold Adjustment

To isolate the bone trabeculae from the background, the contrast threshold was manually adjusted (Threshold) to produce a binary image where trabeculae appeared white against a black background. To complete the binarization process, the “Make Binary” command was applied, enabling clear differentiation between trabeculae and bone marrow.

#### 4.4.2. Bone Density Analysis

Within each ROI, bone density was assessed by measuring the mean gray value, which was interpreted as an indicator of bone mineral density. The measurement was repeated in triplicate for all preoperative, postoperative, and follow-up images [32]. The trend in gray value over time varied depending on the type of lesion treated (aneurysmal bone cyst, simple bone cyst, fibroma, chondroblastoma, or osteosarcoma), but a general pattern was observed:

- Preoperative: low mean gray value, consistent with the radiolucent nature of bone lesions (dark gray appearance).
- Immediate postoperative: slight increase in gray value, due to the presence of the scaffold material, which has a density higher than the lesion but lower than healthy bone.
- Early follow-up (1–2 months): further increase in gray value, indicating the onset of bone mineralization.
- Advanced follow-up (3–6 months): marked increase, associated with more evident new bone formation and gray values approaching those of native bone.
- Long-term follow-up (>6 months): stabilization of gray values at levels similar to surrounding healthy bone, indicating graft integration and structural maturation.

#### 4.4.3. Trabecular Thickness Analysis

Trabecular thickness (Tb.Th.) was assessed using the BoneJ2 plugin implemented in the ImageJ software [33,34]. Specifically, the “Slice Geometry” module of BoneJ2 was employed to perform a two-dimensional morphometric analysis on the binarized radiographic images. In the dialog window, the option “2D Analysis” was selected, appropriate for radiographic slice data. Among the output parameters provided, the “Mean Thickness 2D” value was used to estimate the average trabecular thickness within each image slice, based on the assumption that trabeculae maintain a relatively uniform shape. The measurement was repeated in triplicate for all preoperative, postoperative, and follow-up images. The trend in trabecular thickness over time varied depending on the type of lesion treated (e.g., aneurysmal bone cyst, simple bone cyst, fibroma, chondroblastoma, or osteosarcoma), but a general pattern was observed:

- Preoperative: Tb.Th. values were low, reflecting bone destruction caused by the lesion.
- Immediate postoperative: a slight increase in Tb.Th. was noted, though still markedly below physiological levels.
- Early follow-up (2–6 months): a progressive and significant increase in Tb.Th. was observed, corresponding to the deposition of new bone matrix.
- Advanced follow-up (6–12 months): Tb.Th. continued to increase, with more pronounced trabecular formation.
- Long-term follow-up (>12 months): Tb.Th. values stabilized, reaching levels comparable to those of healthy bone, indicating successful graft integration and bone maturation.

## 5. Conclusions

In conclusion, despite the limited number of patients and the retrospective nature, this study documents one of the first clinical experiences with the use of SmartBone^®^ ORTHO in pediatric oncologic patients. The results pose in favor of confirming known behavior of the investigated device: in this set of pediatric cases, the biomaterial is indeed able to promote prompt osteointegration and progressive remodeling, ensuring mechanical stability and functional recovery. Its good biological tolerance, the absence of major complications, and the ability to adapt to different skeletal sites make it a particularly attractive option in the delicate setting of pediatric oncology, where the preservation of function and skeletal growth is an absolute priority.

Despite the limitations of this retrospective series and its relatively short follow-up, the findings support that SmartBone^®^ ORTHO represents a valid alternative or complement to conventional grafts in the reconstruction of post-resection bone defects in pediatric oncology.

## Figures and Tables

**Table 1:** Clinical characteristics, diagnosis, surgical procedures, and outcomes of the pediatric patients treated with SmartBone^®^ ORTHO.

Code	Age	Sex	Diagnosis and Location	Surgical Intervention SmartBone Type	2nd Surgical Intervention Diagnosis	2nd Surgical Intervention SmartBone Type
ONCO-001	9	M	Bone cyst + pathological fracture, Proximal humerus	Curettage + 2 SmartBone sticks (35 × 4 × 3) + 1 block (15 × 30 × 50)	Recurrence	Curettage + removal + allograft
ONCO-003	11	M	Bone cyst + pathological fracture, Proximal humerus	Curettage + 2 SmartBone blocks (15 × 30 × 50) + Bioglass	No	–
ONCO-006	12	F	Non-ossifying fibroma + fracture, Proximal tibia	Curettage + 1 SmartBone block (15 × 30 × 50)	No	–
ONCO-007	15	M	Aneurysmal bone cyst, Distal fibula	Curettage + 2 SmartBone blocks (15 × 30 × 60)	No	–
ONCO-032	16	M	Simple bone cyst, Calcaneus	Curettage + SmartBone block (15 × 30 × 50) + Bioglass	No	–
ONCO-033	16	F	Aneurysmal bone cyst, Pubic ramus/acetabulum	Curettage + SmartBone block (15 × 30 × 60) + Bioglass	No	–
ONCO-034	17	M	Aneurysmal bone cyst, Proximal tibia	Curettage + SmartBone granules (5.5 g) + Spongostan^®^	Recurrence	Curettage + SmartBone block (15 × 30 × 50)
ONCO-035	15	M	Non-ossifying fibroma, Proximal tibia	Curettage + 2 SmartBone granule boxes (5.5 g)	No	–
ONCO-036	17	F	Aneurysmal bone cyst, Distal femur	Curettage + 2 SmartBone blocks (15 × 30 × 50) + 1 wedge (40 × 25 × 14)	No	–
ONCO-040	16	M	Chondroblastoma, Proximal humerus	Curettage + 2 SmartBone granule boxes + 1 block	Recurrence	Curettage + SmartBone (same config.)
ONCO-041	16	M	Bone cyst, Distal humerus	Curettage + 2 SmartBone blocks (14 × 12 × 12)	No	–
ONCO-044	16	M	Bone cyst, Iliac wing	Curettage + 2 SmartBone rods (35 × 4 × 3) + morselized block	No	–

The table summarizes demographic data (age and sex), diagnosis and anatomical site of the lesion, type of surgical intervention performed, configuration of the SmartBone^®^ ORTHO graft used, and any subsequent surgical revisions. Cases of recurrence are indicated together with the type of secondary intervention and graft employed, when applicable.

## Data Availability

The datasets generated and analyzed during the current study are available from the corresponding author on reasonable request.
